# Sensorimotor gating and clinical outcome following cognitive behaviour therapy for psychosis

**DOI:** 10.1016/j.schres.2011.11.020

**Published:** 2012-02

**Authors:** Veena Kumari, Preethi Premkumar, Dominic Fannon, Ingrid Aasen, Satya Raghuvanshi, Anantha P. Anilkumar, Elena Antonova, Emmanuelle R. Peters, Elizabeth Kuipers

**Affiliations:** a Department of Psychology, Institute of Psychiatry, King's College London, London, UK; b NIHR Biomedical Research Centre for Mental Health, South London and Maudsley NHS Foundation Trust, London, UK; c Division of Psychology, Nottingham Trent University, Nottingham, UK; d University College London Medical School, University College London, London, UK; e South London and Maudsley NHS Foundation Trust, London, UK

**Keywords:** Prepulse inhibition, Startle, Schizophrenia, Symptoms, Antipsychotics, Medication resistance

## Abstract

**Background:**

Prepulse inhibition (PPI) of the startle response refers to the ability of a weak prestimulus to transiently inhibit the response to a closely following strong sensory stimulus. PPI provides an operational index of sensorimotor gating and is reduced, on average, in people with schizophrenia, relative to healthy people. Given the variable response to Cognitive Behaviour Therapy for psychosis (CBTp) and positive associations between pre-therapy brain and cognitive functions and CBT outcome across disorders, we examined whether pre-therapy level of PPI is associated with clinical outcome following CBTp.

**Method:**

Fifty-six outpatients stable on medication with at least one distressing symptom of schizophrenia and willing to receive CBTp in addition to their usual treatment were assessed on acoustic PPI. Subsequently, 28 patients received CBTp (CBTp + treatment-as-usual, 23 completers) for 6–8 months and 28 continued with their treatment-as-usual (TAU-alone, 17 completers). Symptoms were assessed (blindly) at entry and follow-up.

**Results:**

The CBTp + TAU and TAU-alone groups did not differ demographically, clinically or in PPI at baseline. The CBTp + TAU group showed improved symptoms relative to the TAU-alone group, which showed no change, at follow-up. Pre-therapy PPI level correlated positively with post-CBTp symptom improvement.

**Conclusions:**

Relatively intact sensorimotor gating is associated with a good clinical response following a 6–8 months course of NICE compliant CBTp in schizophrenia. Pharmacological or psychological interventions capable of improving PPI may enhance the effectiveness of CBTp in people with schizophrenia, particularly in those who fail to show clinical improvement with currently available antipsychotic drugs and adjunctive CBTp.

## 1 Introduction

Despite marked symptom improvement with the use of antipsychotics in acutely ill patients with schizophrenia (Kasper, 2006), the long-term outcome for up to 40% of patients remains unsatisfactory as they continue to suffer from one or more distressing symptoms despite remaining medication compliant (Conley and Kelly, 2001; Potkin et al., 2009). Additional benefits of cognitive behaviour therapy for psychosis (CBTp) have been reported for such patients (reviews, Pilling et al., 2002; Zimmermann et al., 2005; Pfammatter et al., 2006; Wykes et al., 2008), and symptom improvement may continue even after therapy is terminated (Sensky et al., 2000; Sarin et al., 2011). CBTp is now recommended for the treatment of psychosis in both the National Institute for Health and Clinical Excellence (NICE) updated guidelines in the UK (NICE, 2009) and the Schizophrenia Patient Outcomes Research Team (PORT) Treatment Recommendations in the US (Dixon et al., 2010). The beneficial effects of CBTp, however, are seen with modest effect sizes and to a meaningful degree in only about 50% of patients who undergo this therapy (Pilling et al., 2002; Pfammatter et al., 2006; Wykes et al., 2008). Uncovering the determinants of effective CBTp may a) help to maximise its benefits by targeting the most relevant population, and b) to identify methods to help those who do not show a sufficient response with current antipsychotic medications and CBTp.

A number of studies have focussed on specific predictors of clinical response to CBTp (Garety et al., 1997; Kumari et al., 2009, 2010; Penades et al., 2010; Premkumar et al., 2010, 2011). Cognitive flexibility about delusions (Garety et al., 1997), better cognitive insight (Perivoliotis et al., 2010), and lower conviction scores (Brabban et al., 2009) have all been found to be predictors of a good outcome on delusional thinking. More recently, Penades et al. (2010) reported a positive association between verbal memory and clinical outcome following CBTp. Although we did not find a direct association between symptom improvement following CBTp and pre-therapy cognitive performance, assessed with a number of tests commonly employed in schizophrenia research (Premkumar et al., 2011), we did observe greater hippocampal grey matter volume in CBTp responders, compared to CBTp non-responders in the same sample (Premkumar et al., 2009). This latter finding can be regarded consistent with that of Penades et al. (2010), given the positive association between hippocampal volume and memory in schizophrenia observed across a number of previous studies (review, Antonova et al., 2004). Furthermore, our functional magnetic resonance imaging (MRI) studies have demonstrated that pre-therapy brain activity and functional connectivity between brain regions involved in cognitive flexibility and self-other distinction predict clinical outcome following CBTp (Kumari et al., 2009, 2010). Specifically, we found a positive association between CBTp responsiveness and dorsolateral prefrontal cortex (DLPFC) activity and its connectivity with the cerebellum (Kumari et al., 2009), most likely mediated by PFC–cerebellum contributions to executive processing (Bellebaum and Daum, 2007).

The present study aims to further advance this field by investigating the relationship between pre-CBTp level of sensorimotor gating function, as assessed by prepulse inhibition (PPI) of the acoustic startle response, and the clinical outcome following CBTp. PPI refers to a response reduction in reaction to a strong startling stimulus, ‘pulse’, if this is preceded shortly by a prestimulus, ‘prepulse’, too weak to evoke a measurable startle response itself (Graham, 1975). PPI provides an operational index of sensorimotor gating: while information processing resources are targeted at the prepulse, any incoming information (i.e. the pulse) is attended to at reduced level, thereby protecting the processing of the initial stimulus (i.e. the prepulse) (Geyer et al., 1990). Since the first demonstration by Braff et al. (1978), a large number of studies have shown reduced PPI, on average, in people with schizophrenia (e.g. those reviewed in Braff et al., 2001; Meincke et al., 2004; Swerdlow et al., 2006; Kumari et al., 2007), especially in those who have thought disorder (Perry and Braff, 1994; Perry et al., 1999), hear uncontrollable voices (Kumari et al., 2008a) or have poor global functioning (Swerdlow et al., 2006). Some studies also report small-to-moderate positive associations between reduced PPI and poor performance on measures of attention (Karper et al., 1996; Kumari et al., 2007) and executive function, in particular cognitive flexibility (Butler et al., 1991; Kumari et al., 2007), in schizophrenia, suggesting that deficient gating may interfere with higher order cognitive function. Given these observations, and previous findings indicating that relatively intact (pre-therapy) executive processing is associated with good clinical responsiveness to CBT across many disorders, including depression (Moorey et al., 2001; Julian and Mohr, 2006) and generalized anxiety disorder (Mohlman and Gorman, 2005), we hypothesised that there would be a positive association between pre-therapy PPI level and clinical response to CBTp in patients with schizophrenia. In addition, we explored the relationship between pre-therapy level of startle habituation and CBTp response. Reduced habituation has been found in people with schizophrenia (e.g. Geyer and Braff, 1982; Braff et al., 1992; Takahashi et al., 2008); it is thought to reflect their inability to ignore the repetitive functionally insignificant stimuli and to result in sensory overload (Geyer and Braff, 1987; Geyer et al., 1990).

## 2 Methods

### 2.1 Participants and design

The study included 56 outpatients, 54 with paranoid schizophrenia and 2 with schizoaffective disorder, who were willing to receive 6–8 months of CBTp in addition to the treatment and care they were already receiving from mental health professionals. The clinical diagnosis was made by a trained psychiatrist using the Structured Clinical Interview for DSM-IV (SCID; First et al., 2002). Of 56 patients, 28 received CBTp for 6–8 months in addition to their usual treatment (CBTp + TAU group) and 28 continued to receive treatment as usual (TAU-alone group) during the course of this investigation. This investigation has been carried out as part of a larger project examining MRI, neuropsychological and clinical predictors and correlates of responsiveness to CBTp. The sample of patients included in this report thus overlaps with the samples examined in our recent reports (Kumari et al., 2009, 2010, 2011; Premkumar et al., 2009, 2010, 2011). However, none of these published reports examined or reported any psychophysiological (startle) data.

The patients in the CBTp + TAU and TAU-alone groups were recruited from the same geographical area (South London, UK) and were identified by their local treating psychiatric consultants as suitable for CBTp. All included patients were required to be on stable doses of antipsychotics for ≥ 2 years and on the present antipsychotic for > 3 months, to receive a rating of ≥ 60 on the Positive and Negative Syndrome scale (PANSS) (Kay et al., 1987), and to have at least one persistent positive symptom (a score of 3 or above on at least one of the positive symptoms items of the PANSS, which they experienced as distressing).

As described in Kumari et al. (2011), the recruitment of patients and the creation of study groups followed a cohort case-controlled design. It involved the following steps: (i) a patient referred by his/her local consultant and accepted for CBTp by the Psychological Interventions Clinic for Outpatients with Psychosis (PICuP), South London and Maudsley NHS Foundation Trust, (ii) study introduced to the patient by PICuP staff, (iii) patient contacted by a member of the research team if interested in taking part, (iv) if found suitable, patient recruited as part of the CBTp + TAU group, and (v) another patient with similar demographic and clinical characteristics (to that of the patient included in the CBTp + TAU group) recruited for the TAU-alone group via local consultants and studied over the same interval as the CBTp + TAU group. With the resources then available to the South London and Maudsley NHS Foundation Trust, out of all patients potentially eligible for CBTp, only around 10% patients were referred for CBTp. There were no explicit biases in which patients received CBTp. Allocation of CBTp was driven by clinical resource limitations of the NHS Trust and not by patient characteristics. The researchers were independent of clinical decisions about which patients were referred for CBTp. All patients underwent clinical assessment at entry and follow-up (6–8 months later).

Of 56 patients, 3 (1 CBTp + TAU, 2 TAU-alone) did not provide usable startle data. Of remaining 53 patients, 23 patients of the CBTp + TAU group and 17 patients of the TAU-alone group completed follow-up clinical assessment, and had remained on the same type and dosage of antipsychotic medication during the follow-up period. Table 1 presents clinical and demographic characteristics of the final sample.

The study was approved by the research ethics committee of the Institute of Psychiatry and the South London and Maudsley NHS Foundation Trust. All participants provided written informed consent after the study procedures had been explained to them.

### 2.2 Cognitive behaviour therapy for psychosis (CBTp) and treatment-as-usual (TAU) procedures

After baseline clinical and PPI assessments, the CBTp + TAU group received 6–8 months of CBTp following the procedures described in a published manual (Fowler et al., 1995). Therapy sessions were conducted weekly or fortnightly, as preferred by the patient, for up to one hour. Patients received an average of 16 sessions, as recommended by NICE guidelines in the UK (NICE, 2009), at the PICuP clinic. The therapists were supervised by one of the two investigators (EK, ERP) who have extensive experience of CBTp in schizophrenia. PICuP has evidence of good therapy outcomes (Peters et al., 2010).

TAU provided to all patients prior to, and during, the investigation consisted of management offered by a case management team with a dedicated care-coordinator who saw the patient on a regular basis, in addition to a psychiatrist and other specialists, such as a benefits adviser or a vocational advisor, as required.

### 2.3 Symptom assessment

All patients were rated, using the PANSS (Kay et al., 1987), at entry (baseline) and then 6–8 months later (follow-up) by an experienced psychiatrist (DF), who had no role in patient recruitment or clinical management of the recruited patients and was blind to whether patients received CBTp or not. Appointments for these assessments were made by another member of the research team.

### 2.4 PPI assessment: paradigm and procedure

A commercially available human startle response monitoring system (Mark II, SR-Lab, San Diego, California) was used to generate and deliver the acoustic stimuli, and to record and score the electromyographic (EMG) activity for 250 ms starting from the onset of the acoustic startle stimulus. Acoustic stimuli were presented to study participants binaurally through headphones. The pulse-alone stimulus was a 40-ms presentation of 114-dB (A) white noise and the prepulse stimulus a 20-ms presentation of 85-dB (A) white noise, both over 70-dB (A) continuous background noise. The session began with a 5 min acclimatization period consisting of 70 dB (A) continuous white noise. During the experiment, participants received four blocks of 12 trials each, after an initial pulse alone trial; each block consisted of three pulse alone trials, three prepulse trials with a 30-ms prepulse-to-pulse (onset-to-onset) interval, three prepulse trials with a 60-ms prepulse-to-pulse interval, and three prepulse trials with a 120-ms prepulse-to-pulse interval presented to participants in a pseudorandom order with a mean inter-trial interval of 15 s (range 9–23 s).

The experimental procedures for recording and scoring the startle reflexes were identical to those reported previously (e.g. Kumari et al., 2008a, 2008b). Eye blink component of the startle was indexed by recording EMG activity of the orbicularis oculi muscle directly beneath the right eye, by positioning two miniature silver/silver chloride electrodes. Recorded EMG activity was band-pass filtered, as recommended by the SR-Lab. A 50-hz filter was used to eliminate the 50-Hz interference. The EMG data were at first inspected on a trial-by-trial basis offline and then scored, blind to group membership, using the analytic programme of this system for response amplitude (in arbitrary analogue-to-digital units) and latencies to response onset and peak. Responses (< 5%) were rejected if the onset and peak latencies differed by more than 95 ms or when the baseline values shifted by more than 50 units. PPI was computed for each participant separately for each trial type as (a − b / a) × 100, where “a” = pulse-alone amplitude and “b” = amplitude over prepulse trials. Percent of PPI, rather than absolute amount of PPI (i.e. arithmetic difference between pulse-alone and prepulse trials), was used since this procedure eliminates the influence of individual differences in startle responsiveness.

Participants were told that the experiment was to measure their reaction to a number of noise-bursts, but no specific instructions were given on whether to attend or ignore them. They were requested to keep their eyes open during the experiment. There was no explicit restriction on smoking intake prior to testing but care was taken not to start PPI experiment for at least 20 min after a patient smoked a cigarette, in order to prevent a state of smoking withdrawal or a heavy intake during the testing session that may transiently affect PPI (Kumari et al., 2001).

### 2.5 Data analysis

#### 2.5.1 Baseline group comparisons

Possible group differences between the final CBTp + TAU and TAU-alone groups in demographic (age, years in education) and clinical variables (age at illness onset, baseline PANSS scores, medication dose) were analysed using independent sample t-tests. Possible group differences in the amplitude and habituation of the startle response over pulse-alone trials were examined by a 2 (Group: CBTp + TAU, TAU-alone) × 4 (Block: 4 blocks, each consisting of three trials) ANOVA, with Group as a between-subjects and Block as a within-subjects factor. To examine group differences in PPI, PPI (%) scores were subjected to a 2 (Group, as above) × 3 (Trial type: 30-ms, 60-ms and 120-ms prepulse trials) ANOVA, with Group as a between-subjects and Trial type as a within-subjects factor. Latencies to response peak were analysed with a 2 (Group) × 4 (Trial Type: pulse-alone and 3 prepulse trials) ANOVA with Group as a between-subjects and Trial Type as a within-subjects factor. Habituation, PPI and latencies to response onset were initially analysed with Sex as an additional between-subjects factor but no main effect of Sex was found and Sex did not interact with Group, Block (habituation) and Trial Type (PPI and latencies to response peak) for any of the variables. This (Sex) factor was therefore dropped from the ANOVAs.

#### 2.5.2 Clinical effects of CBTp+TAU vs TAU-alone

The change in symptoms from baseline to follow-up was investigated using a Group (CBTp + TAU, TAU-alone) × Time (baseline, follow-up) ANOVA with Group as a between-subjects factor and Time as a within-subjects factor. A significant Group × Time effect was followed up by paired t-tests on total and sub-scale PANSS scores separately in the CBTp + TAU and TAU-alone groups. Following the observation of significant symptom reduction in the CBTp + TAU group, but not in the TAU-alone group, we confirmed the effects of CBTp using ANCOVAs on symptom change scores (baseline minus follow-up) co-varying for baseline symptoms.

#### 2.5.3 Association between pre-therapy PPI and symptom improvement following CBTp

For this analysis, in addition to symptom change scores, we also computed the degree of change in symptoms as a function of initial severity, i.e. the residual change in symptoms, by regressing the initial PANSS (total and sub-scales) scores on follow-up scores, and included this as a further outcome measure of CBT responsiveness following the method used by Siegle et al. (2006). The association between PPI and symptom improvement following CBTp (both absolute and residual symptom change) was examined using Pearson's correlations. Such correlations were then also performed in the TAU-alone group and the strength of the significant correlation between PPI and total symptom change in the two groups was compared using Fisher's z.

#### 2.5.4 Association between pre-therapy startle amplitude and habituation and symptom improvement following CBTp

Possible associations between the amplitude (mean amplitude over all pulse-alone trials) and habituation of the response (computed as % reduction in amplitude from the first block, to the last block, of pulse-alone trials) with symptom improvement following CBTp were examined using Pearson's correlations. Such correlations were also performed in the TAU-alone group, and the strength of the correlation between habituation and total symptom change in the two groups was compared using Fisher's z.

All analyses were performed by SPSS windows (version 16). Alpha level for testing significance of effects was p = 0.05 unless stated otherwise.

## 3 Results

### 3.1 Baseline group comparisons

As shown in Table 1, the final CBTp + TAU and TAU-alone groups, after the loss of some patients from each group, did not differ in age, education, IQ, age at illness onset, baseline symptoms, antipsychotic dose (all p values > 0.15) .

The two groups showed comparable amplitude and habituation of the startle response over pulse-alone trials as demonstrated by a significant main effect of Block [F (3, 114) = 14.77, p < 0.001] indicating habituation of the startle response over four blocks [Linear F (1, 38) = 18.60, p < 0.001] in both groups, but no significant Group [F (1,37) = 2.25, p = 0.14] or Group × Block effect [F (1,111) = 0.52, p = 0.70]. For PPI too, there was no effect of Group [F (1,38) = 0.01; p = 0.96] indicating comparable PPI in the two groups. There was, as found in previous PPI studies, a significant main effect of Trial type [F (2, 76) = 16.18, p < 0.001], reflecting a linear increase in PPI from 30-ms through 60-ms to 120-ms prepulse trials [Linear F (1, 38) = 34.41, p < 0.001], but no Group × Trial Type interaction [Group × Trial type: F (2, 76) = 1.45, p = 0.24]. Finally, the two groups did not differ in the latencies to peak over the four trial types as there was only a main effect of Trial Type [F (3, 114) = 3.86, p = 0.01] and no Group [Group: F (1,38) = 1.58, p = 0.22] or Group × Trial type effect [F (1,114) = 1.91, p = 0.13]. Mean (s.e.m) startle amplitudes for blocks 1–4, PPI for 30-ms, 60-ms, and 120-ms trials, and mean latencies to peak over the pulse-alone and prepulse trials in the CBTp + TAU and TAU-alone groups are presented in Table 2.

### 3.2 Clinical effects of CBTp+TAU vs TAU-alone

As expected given our previous reports from overlapping samples (Kumari et al., 2009, 2010, 2011; Premkumar et al., 2009, 2010, 2011), CBTp + TAU, but not TAU-alone, patients showed changes in symptoms from baseline to follow-up [Group × Time: total PANSS scores, F = 7.48, df = 1,38, p = 0.009; positive symptoms, F = 4.94, p = 0.03; negative symptoms, F = 6.43, p = 0.02; general psychopathology: F = 4.25, p = 0.04) (Table 1). Only the CBTp + TAU group showed reduced symptoms at follow-up (total PANSS scores: t = 3.65, df = 22, p = 0.001; positive symptoms: t = 4.42, p < 0.001; negative symptoms: t = 2.51, p = 0.02; general psychopathology: t = 2.80, p = 0.01).

CBTp + TAU versus TAU-alone group differences in symptom improvement (change scores) remained significant after co-varying for baseline symptoms (total PANSS scores: F = 9.92, df = 1,37, p = 0.003; positive symptoms: F = 7.51, p = 0.009; negative symptoms: F = 10.78, p = 0.002; general psychopathology: F = 6.86, p = 0.01). The number of years in education or illness duration was not associated with CBTp responsiveness (p values > 0.40).

### 3.3 Association between pre-therapy PPI and symptom improvement following CBTp

**Fig. 1:**
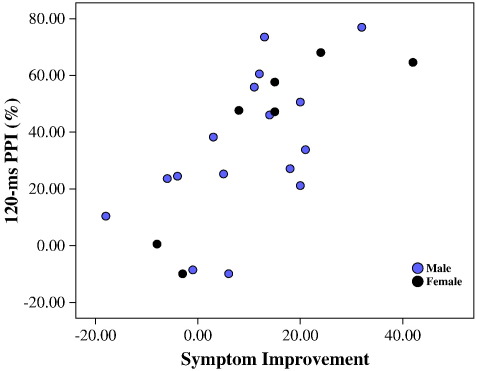
Scatter plot of 120-ms PPI across PANSS symptom improvement (baseline total score minus follow-up total score) in the CBTp + TAU group.

Symptom improvement (absolute change) following CBTp for PANSS total, negative, and general psychopathology scores was significantly positively associated with mean PPI (across 30-ms, 60-ms and 120-ms trials), and with 120-ms PPI when examined separately at the three intervals (Table 3, Fig. 1). The positive correlation between improvement following CBTp for PANSS positive scores symptoms and pre-therapy PPI failed to reach formal significance. However, the strength of this (pre-therapy PPI — positive symptom improvement) correlation was not significantly weaker than that observed for the PANSS negative and general psychopathology scores and the strongest correlation occurred for the PANSS total scores, indicating that pre-therapy PPI was associated with post-CBTp improvement in PANSS symptoms across all three subscales, rather than on particular PANSS subscales. Residual symptom reduction was also positively associated with 120-ms PPI only (Table 3). No significant correlations occurred in the TAU-alone group. The strength of the correlation between pre-therapy PPI and total symptom change in the CBTp + TAU group was stronger than that observed in the TAU-alone group (absolute symptom change, Fisher's z = 2.14, p = 0.03; residual symptom change, Fisher's z = 1.85, p = 0.06). Finally, although this study does not allow a meaningful analysis of possible sex-specific associations between PPI and response to CBTp due to insufficient number of women, the positive PPI-CBTp response associations, especially with 120-ms PPI, appear to be present in both sexes (Table 3, Fig. 1).

### 3.4 Association between pre-therapy startle amplitude and habituation and symptom improvement following CBTp

The mean pulse-alone amplitude did not correlate significantly but startle habituation showed a positive association with CBTp response as assessed with reduction from baseline to follow-up in the total PANSS scores (for improvement in total PANSS symptoms, r = 0.430, p = 0.04; positive symptoms, r = 0.361, p = 0.09; negative symptoms, r = 0.307, p = 0.15; general psychopathology, r = 0.395, p = 0.06). No correlation between the amplitude or habituation and clinical change at follow-up was found in the TAU-alone group (total PANSS symptoms, r = − 0.08; positive symptoms, r = 0.11; negative symptoms, r = − 0.012; general psychopathology, r = 0.012). The correlation between pre-therapy habituation and total symptom change in the CBTp + TAU group was non-significantly different from that observed in the TAU-alone group (Fisher's z = 1.55, p = 0.12); however, near-zero correlation in the TAU-alone group and significantly positive association in the TAU + CBTp may argue for a small but true association between the rate of habituation and CBTp response.

## 4 Discussion

Supporting our hypothesis, the finding of this study revealed a positive association between (pre-therapy) PPI and symptom improvement following CBTp. The PPI level seen in patients of both the CBTp + TAU and TAU-alone groups, on average, was markedly low relative to the level of PPI observed in healthy people [reported in Kumari et al., 2008a; mean (s.e.m) 30-ms PPI = 17.44% (5.12), 60-ms = 30.75% (4.85), 120-ms = 45.86% (5.24)] who had been tested using the same paradigm and procedure in parallel to the patients included in this report. As mentioned earlier (Introduction), there is a sizable proportion of patients who, despite receiving and being compliant with currently available antipsychotic therapies and having received adjunctive CBTp, continue to suffer from distressing symptoms. The present finding suggests that this sub-group of patients is characterised by disturbed sensorimotor gating function. Those with relatively intact gating may be better able to filter out internal stimuli (Kumari et al., 2008a) and engage more fully during therapy sessions, and in turn show a better response to CBTp. The inability to suppress intrusive memories, thoughts and other internal stimuli is considered to be the behavioural correlates of deficient PPI across psychiatric disorders (Geyer, 2006).

The relationship between PPI and clinical responsiveness to CBTp in this study, however, was significantly evident only at 120-ms prepulse-to-pulse intervals. The relationship with 30-ms and 60-ms PPI, although in the same direction, failed to reach statistical significance. There could be two reasons for this. The first that there was simply less power available to detect CBTp response-PPI association at 30-ms and 60-ms intervals due to a limited range of PPI scores especially at 30-ms interval. The second possibility is that cognitive processes involved in PPI at 120-ms interval, as discussed below, are particularly relevant to effective CBTp.

PPI is considered primarily to be a pre-attentive mechanism but it may be susceptible, especially at > 60 ms prepulse-to-pulse intervals, to cognitive processes controlled in a ‘top down’ manner by the cortex (Hazlett and Buchsbaum, 2001). While active attention to prepulses is not necessary for PPI to occur (Bluementhal, 1999), actively ‘attended’ prepulses produce more PPI than the ‘ignored’ ones, at prepulse-to-pulse intervals greater than 60 ms (Dawson et al., 1997; Filion and Poje, 2003; Jennings et al., 1996; Schell et al., 2000). Studies in schizophrenia samples, which assessed PPI at longer than 60 ms interval or examined mean PPI across short and long intervals, have also shown small-to-modest positive association between PPI and performance on the Wisconsin Card Sort Test, which is widely regarded as a measure of planning and strategy formation (Butler et al., 1991; Kumari et al., 2007), and negative correlation with distractibility on the Continuous Performance Test (Karper et al., 1996; Kumari et al., 2007). Recent studies in healthy subjects have also found greater PPI in those with superior performance on DPFC-based tasks of planning, strategy formation, and selective attention (Bitsios and Giakoumaki, 2005; Bitsios et al., 2006; Giakoumaki et al., 2006). We previously reported a positive association between DLPFC grey matter volumes and 120-ms PPI in schizophrenia (Kumari et al., 2008b) and stronger improvement in PANSS symptoms following CBTp in patients with greater DPLFC activity (Kumari et al. , 2009).

Our finding may be taken as further evidence that brain areas involved in the top-down processing of information are associated with CBTp responsiveness (van der Gaag, 2006). However, our study is limited by a relatively small sample size, and thus requires replication. Further studies also need to examine whether the observed association between higher pre-therapy PPI and a good outcome following CBTp is specific to this particular therapy, or extends to clinical outcomes following other psychological interventions. In a study by Granholm et al. (2008), cognitive deficits predicted poorer function overall in older psychotic patients but did not moderate the effect of cognitive behavioural social skill training (CBSST). The therapy in their study, however, was targeted specifically at social functioning rather than at distress and symptom reduction.

This study also found a small association between the rate of startle habituation and response to CBTp, despite the fact that it utilised a relative brief experiment with only 12 pulse-alone stimuli and thus was not specifically designed to probe such an association. This finding is complementary to the earlier discussed positive association between PPI and CBTp response and confirms that a reduced ability to prevent sensory overload is associated with a poor response to CBTp.

The finding of this study, despite some limitations, may have practical implications. Recent attempts to discover new drugs for patients with schizophrenia who remain distressed by their symptoms despite receiving and being compliant with current antipsychotic medications have met with limited (if any) success. As suggested very recently by Swerdlow (2011), a more meaningful progress in this field may be made by focusing on targets which can enhance the efficacy of psychological therapies in this population. It would be valuable, for example, to examine whether medication which can enhance PPI (reviews, Swerdlow et al., 2008; Swerdlow, 2011), or psychological interventions, such as attention training, which may be able to improve, at least to some extent, attentional modulation of PPI (Scholes and Martin-Iverson, 2010), help to maximise benefit from CBTp in such patient groups. In this context, psychophysiological measures, such as PPI, with high test-retest reliability (review, Swerdlow et al., 2008) and preferably low measurement error may provide a more sensitive tool than commonly used neuropsychological tests (often involving multiple cognitive domains). Neuropsychological tests appear less sensitive than PPI (and MRI) as predictors of CBTp outcome (i.e. failed to relate to CBTp response in a highly overlapping sample; Premkumar et al., 2011).

## Role of funding source

The sponsors had no role in study design; in the collection, analysis and interpretation of data; in the writing of the report; or in the decision to submit the paper for publication.

## Contributors

Veena Kumari, Emmanuelle Peters and Elizabeth Kuipers designed the study. Emmanuelle R Peters and Elizabeth Kuipers supervised cognitive behaviour therapy for psychosis. Dominic Fannon and Ananatha PP Anilkumar performed the clinical diagnostic interviews and symptom ratings. Preethi Premkumar, Ingrid Assen, Satya Raghuvanshi and Elena Antonova assisted with data collection, scored and data-based clinical and psychophysiological measures. Veena Kumari undertook statistical analysis and wrote the first draft of the manuscript. All authors contributed to the final version.

## Conflict of interest

The authors declare no conflict of interest.

## Figures and Tables

**Table 1:** Demographics, task performance, and clinical characteristics of participants.

	CBTp + TAU group (n = 23, 16 men and 7 women)		TAU-alone group (n = 17, 14 men and 3 women)	
	Mean (S.D.)	Mean (S.D.)	Mean (S.D.)	Mean (S.D.)
Demographics	Baseline	Follow-up	Baseline	Follow-up
Age (years)	36.00 (7.66)		39.53 (9.15)	
Education (years)	13.78 (2.81)		13.35 (1.54)	
Predicted IQ^a^	109.13 (8.14)		106.71 (9.81)	
Age at illness onset (years)	23.74 (7.98)		25.65 (8.31)^b^	
PANSS symptoms				
Positive symptoms	18.35 (5.16)	c15.04 (4.57)	18.71 (3.22)	18.06 (3.09)
Negative symptoms	18.22 (4.88)	c15.91 (4.89)	19.53 (3.97)	20.82 (4.17)
General psychopathology	33.35 (7.79)	c28.56 (7.86)	34.65 (4.87)	34.64 (6.12)
Total symptoms	69.91 (14.87)	c59.52 (16.22)	72.88 (9.26)	73.52 (11.36)
Antipsychotic medication type	20 patients on atypical; 3 on both atypical and typical antipsychotics	As baseline	14 patients on atypical; 3 on both atypical and typical antipsychotics	As baseline
Antipsychotic dose in chlorpromazine equivalents (mg)	521.07 (387.95)		517.33 (344.32)	

a National Adult Reading Test (Nelson and Willison, 1991).

b PANSS: Positive and Negative Symptom Scale (Kay et al., 1987).

c Symptom reduction (p < 0.05) in the CBTp + TAU, relative to the TAU-alone (no significant change in TAU-alone group, p > 0.05) group, at follow-up relative to baseline.

**Table 2:** Mean (standard error of the mean, s.e.m.) response amplitudes over the four blocks of three pulse-alone trials each, PPI and latencies to response peak in CBTp + TAU and TAU-alone patients.

Startle amplitude (analogue-to-digit units)	CBTp + TAU group (n = 23, 16 men and 7 women)	TAU-alone group (n = 17, 14 men and 3 women)
	Mean (s.e.m.)	Mean (s.e.m.)
Block 1	267.15 (39.61)	229.88 (56.77)
Block 2	211.22 (30.19)	130.09 (23.74)
Block 3	188.69 (31.13)	116.76 (19.49)
Block 4	154.04 (24.00)	101.07 (18.59)

PPI (%)		
30-ms	8.97 (4.93)	7.69 (10.33)
60-ms	16.38 (5.36)	23.56 (6.08)
120-ms	35.86 (5.58)	28.74 (9.53)

*Latencies to response peak (ms)*		
Pulse-alone	68.57 (1.25)	68.67 (2.29)
30-ms	62.09 (1.49)	67.39 (2.27)
60-ms	60.50 (1.61)	65.59 (3.28)
120-ms	66.78 (2.31)	66.46 (3.09)

**Table 3:** Correlations (Pearson's r) between pre-therapy PPI levels and symptom improvement at follow-up.

PANSS symptom improvement	CBTp + TAU group (n = 23; 16 men and 7 women)				TAU-alone group (n = 17, 14 men and 3 women)			
	Mean PPI (across 30-, 60-, 120 ms)	30-ms PPI	60-ms PPI	120-ms PPI	Mean PPI (across 30-, 60-, 120 ms)	30-ms PPI	60-ms PPI	120-ms PPI
*Absolute change*								
Positive symptoms	0.316 (M: 0.450; W: 0.141)	0.189	0.270	0.320 (M: 0.325; W: 0.297)	− 0.084	− 0.120	− 0.111	− 0.007
Negative symptoms	0.407⁎ (M: 0.283; W: 0.774⁎)	0.126	0.191	0.665⁎⁎⁎ (M: 0.477#; W: 0.895⁎)	0.143	0.074	0.196	0.149
General psychopathology	0.420⁎ (M: 0.490; W: 0.208)	0.142	0.222	0.652⁎⁎⁎ (M: 0.582⁎; W: 0.761⁎)	0.109	0.093	0.135	0.084
PANSS total	0.466⁎ (M: 0.509⁎;W: 0.378)	0.176	0.266	0.689⁎⁎⁎ (M: 0.583⁎; W:0.845⁎)	0.085	0.035	0.110	0.101

*Residual change*								
Positive symptoms	0.134	− 0.032	0.020	0.326 (M: 0.228; W: 0.467)	− 0.057	− 0.040	− 0.142	− 0.006
Negative symptoms	0.174	− 0.067	− 0.034	0.506⁎ (M: 0.085; W: 0.912⁎⁎)	0.059	0.004	0.193	0.019
General psychopathology	0.222	− 0.018	0.050	0.491⁎ (M: 0.359; W: 0.714)	− 0.006	− 0.008	0.074	− 0.054
PANSS total	0.301	0.033	0.104	0.581⁎⁎ (M: 0.414; W: 0.827⁎)	0.030	0.002	0.084	0.018

M = men; W = women.

# p = 0.06.

⁎ p < 0.05.

⁎⁎ p < 0.01.

⁎⁎⁎ p < 0.001.
